# Structural insights into the effect of active-site mutation on the catalytic mechanism of carbonic anhydrase

**DOI:** 10.1107/S2052252520011008

**Published:** 2020-09-09

**Authors:** Jin Kyun Kim, Cheol Lee, Seon Woo Lim, Jacob T. Andring, Aniruddha Adhikari, Robert McKenna, Chae Un Kim

**Affiliations:** a Department of Physics, Ulsan National Institute of Science and Technology (UNIST), Ulsan 44919, Republic of Korea; b Department of Biochemistry and Molecular Biology, University of Florida, Gainesville, FL 32610, USA

**Keywords:** carbonic anhydrase II, metalloenzymes, active-site mutation, active-site water dynamics, zinc ion, X-ray crystallography, enzyme mechanism, structural biology

## Abstract

Enzymes are catalysts of biological processes. Significant insight into their catalytic mechanisms has been obtained by relating site-directed mutagenesis studies to kinetic activity assays. However, revealing the detailed relationship between structural modifications and functional changes remains challenging owing to the lack of information on reaction intermediates and of a systematic way of connecting them to the measured kinetic parameters. Here, a systematic approach to investigate the effect of an active-site-residue mutation on a model enzyme, human carbonic anhydrase II (CA II), is described. Firstly, structural analysis is performed on the crystallographic intermediate states of native CA II and its V143I variant. The structural comparison shows that the binding affinities and configurations of the substrate (CO_2_) and product (HCO_3_^−^) are altered in the V143I variant and the water network in the water-replenishment pathway is restructured, while the proton-transfer pathway remains mostly unaffected. This structural information is then used to estimate the modifications of the reaction rate constants and the corresponding free-energy profiles of CA II catalysis. Finally, the obtained results are used to reveal the effect of the V143I mutation on the measured kinetic parameters (*k*_cat_ and *k*_cat_/*K*_m_) at the atomic level. It is believed that the systematic approach outlined in this study may be used as a template to unravel the structure–function relationships of many other biologically important enzymes.

## Abstract 2

X-ray crystallography was used to elucidate the effect of a single-site mutation on the activity of a native metalloenzyme. The subtle structural modifications around the active site of the enzyme were correlated with the retarded catalytic efficiency in terms of the mechanistic steps and their kinetics.

## 1. Introduction   

Enzymes greatly enhance the catalytic rates of biochemical reactions compared with their uncatalyzed counterparts and are therefore essential to speed up biochemical processes (Jencks, 1987 ▸; Fersht, 1999 ▸; Frey & Hegeman, 2007 ▸). Enzyme active sites provide highly optimized microenvironments for their specific substrates by providing reactive groups such as nucleophiles or acids/bases that stabilize the transition state. Consequently, changes in the active-site residues can have large effects on enzyme activity. However, direct prediction of the impact of a single mutation on the activity of an enzyme remains challenging owing to the lack of precise correlations between the structure of the protein and its function at atomic resolution (Ishida, 2010 ▸). In this study, we describe the effect of a single amino-acid variation on a prototypical enzyme, human carbonic anhydrase II (CA II), by correlating its high-resolution reaction-intermediate structures with the measured kinetic parameters. Human carbonic anhydrases are well suited to serve as a model system for our study because their structures and active sites are well defined, and their overall enzymatic mechanism is fairly straightforward and has been studied extensively (Krishnamurthy *et al.*, 2008 ▸).

Human carbonic anhydrases catalyze the reversible hydration/dehydration of CO_2_/HCO_3_^−^ (Davenport, 1984 ▸; Christianson & Fierke, 1996 ▸; Chegwidden *et al.*, 2013 ▸; Frost & McKenna, 2013 ▸; Supuran & De Simone, 2015 ▸). In the CO_2_-hydration direction, the first step of catalysis is the conversion of CO_2_ into HCO_3_^−^ via the nucleophilic attack of a zinc-bound hydroxide. This reaction is followed by the displacement of the zinc-bound HCO_3_^−^ by a water molecule (equation 1, where E stands for the enzyme; Silverman & Lindskog, 1988 ▸). The second step involves the transfer of a proton from the zinc-bound water to bulk solvent, regenerating the zinc-bound hydroxide (equation 2, where B stands for a general base: either a water or a proton-shuttling residue).
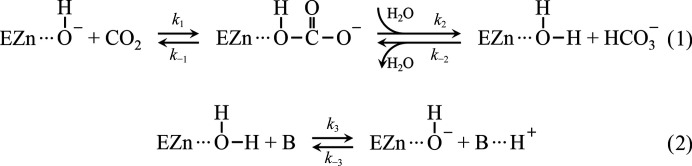


**Figure 1:**
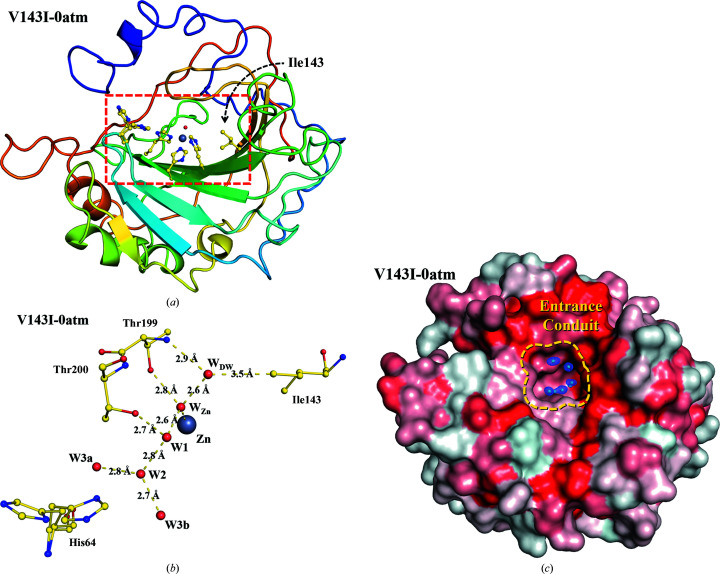
Structure of V143I CA II. (*a*) Overall structure of V143I-0atm: V143I CA II with no CO_2_ pressurization. The active site (red box) is located at a depth of 15 Å from the surface. Note that Ile143 is located at the hydrophobic pocket in the active site. (*b*) Ordered water network in the hydrophilic region serving as a proton-transfer pathway. (*c*) Surface rendition of V143I-0atm. The entrance conduit (diameter of 7–10 Å, guided with a yellow dotted line) connects the active site to the bulk solvent outside, forming the replenishment pathway. The electron density of the entrance-conduit waters is contoured at 1.5σ. Hydrophobic amino acids are shaded in red, while hydrophilic amino acids are coloured white.

In CA II, the active-site zinc is located at the base of a 15 Å deep cleft and is tetrahedrally coordinated by three histidines (His94, His96 and His119) and a zinc-bound water (W_Zn_) [Fig. 1 ▸(*a*)] (Christianson & Fierke, 1996 ▸). The active-site cavity is further subdivided into two distinct faces consisting of hydrophilic and hydrophobic residues. The hydrophilic face (Tyr7, Asn62, His64, Asn67, Thr199 and Thr200) of the active site coordinates the hydrogen-bonded water network (W1, W2, W3a and W3b) that connects the zinc-bound water to His64, the proton-shuttling residue [Fig. 1 ▸(*b*)] (Steiner *et al.*, 1975 ▸; Tu *et al.*, 1989 ▸; Nair & Christianson, 1991 ▸; Fisher *et al.*, 2005 ▸, 2010 ▸; Fisher, Maupin *et al.*, 2007 ▸; Fisher, Tu *et al.*, 2007 ▸; Maupin & Voth, 2007 ▸; Silverman & McKenna, 2007 ▸; Zheng *et al.*, 2008 ▸). It is known that the proton-transfer process is the rate-limiting step in CA II catalysis (Silverman & McKenna, 2007 ▸).

The hydrophobic face (Val121, Val143, Leu198, Val207 and Trp209) is located adjacent to the zinc-bound hydroxide and is responsible for substrate binding (Liang & Lipscomb, 1990 ▸; Domsic & McKenna, 2010 ▸). Leu198, Trp209 and Val121 constitute the mouth and sides of the hydrophobic pocket, while Val143 comprises the base of the hydrophobic pocket. A water molecule termed the ‘deep water’ (W_DW_) is located at the mouth of this pocket and forms van der Waals contacts with Leu198 and Trp209. W_DW_ occupies the pre-catalytic association site for substrate and is displaced by one of the O atoms of CO_2_ during binding (Domsic *et al.*, 2008 ▸). The hydrophobic pocket residues are highly conserved and are known to be critical for CO_2_ sequestration, although they do not directly interact with the CO_2_ molecule.

Between the hydrophilic and hydrophobic sides of the active site, a cluster of ordered waters has recently been identified located near the active-site entrance, termed the entrance-conduit (EC) waters [Fig. 1 ▸(*c*)] (Kim *et al.*, 2018 ▸). This ordered water ensemble connects the active site to the external bulk solvent, creating a pathway where water, substrate and product can interchange and interact with bulk solvent. These EC waters are believed to be involved in water replenishment during catalysis, displacing the zinc-bound bicarbonate and restoring the proton-transfer water network.

In order to elucidate the catalytic details of the active site of CA II, several mutational studies have been performed. Most of these variants have been focused at the zinc ion-binding site (Alexander *et al.*, 1993 ▸; Kiefer *et al.*, 1993 ▸; Ippolito & Christianson, 1994 ▸; Lesburg & Christianson, 1995 ▸; Huang *et al.*, 1996 ▸; Lesburg *et al.*, 1997 ▸), the hydrophilic side (proton-transfer pathway; Behravan *et al.*, 1990 ▸; Krebs, Ippolito *et al.*, 1993 ▸; Xue *et al.*, 1993 ▸; Ippolito *et al.*, 1995 ▸; Huang *et al.*, 2002 ▸; Tu *et al.*, 2002 ▸; Fisher *et al.*, 2005 ▸; Zheng *et al.*, 2008 ▸; Turkoglu *et al.*, 2012 ▸; Mikulski *et al.*, 2013 ▸; Aggarwal *et al.*, 2014 ▸) and the hydrophobic pocket (CO_2_-binding site; Alexander *et al.*, 1991 ▸; Fierke *et al.*, 1991 ▸; Nair *et al.*, 1991 ▸; Krebs, Rana *et al.*, 1993 ▸; West *et al.*, 2012 ▸; Nair & Christianson, 1993 ▸). In the hydrophobic pocket, a series of mutational studies have been performed targeting the Val121, Val143 and Leu198 residues. It was found that CA catalysis is severely compromised (an ∼10^4^–10^5^-fold decrease) when the deep water W_DW_ is displaced by replacement of the relevant amino acid by one with a larger side chain (for example, replacement of Val143 by Phe or Tyr), leading to a substantial blockage of CO_2_ binding. On the other hand, some of the point mutations, such as Val121 to Ala, Val143 to Ile and Leu198 to Glu, do not directly displace W_DW_ and the CA catalysis is only moderately compromised (a fewfold to a 20-fold decrease). Understanding these moderate effects is most challenging as the overall structures and active sites show little deviation when compared with native CA II. It is expected that delicate perturbations are introduced at the level of intermediate structures during the moderately modified CA catalysis.

In this study, we investigate one of the most challenging cases and describe the subtle effects brought on by a Val143 to Ile (V143I) mutation. As shown in Table 1 ▸, the V143I variant shows an approximately tenfold decrease in *k*_cat_/*K*_m_, while *k*_cat_ remains almost the same as that for native CA II. Structural analysis was performed by comparing the catalytic intermediate states of native and V143I CA II, which were obtained by cryocooling protein crystals under four different CO_2_ pressures [ranging from 0 (no CO_2_ pressurization) to 15 atm]. The intermediate states are henceforth referred to as native-0atm (PDB entry 6km3), native-7atm (PDB entry 6km4), native-13atm (PDB entry 6km5) and native-15atm (PDB entry 6km6) and as V143I-0atm (PDB entry 6klz), V143I-7atm (PDB entry 6km0), V143I-13atm (PDB entry 6km1) and V143I-15atm (PDB entry 6km2), respectively. Based upon the structural modifications, we successfully estimated the alterations in the reaction rate constants and the corresponding free-energy profiles in the CA II enzymatic mechanism. This study systematically reveals how a single point mutation influences an enzyme’s catalytic pathway at the atomic level, leading to an estimation of the kinetics governing its individual mechanistic steps.

## 2. Results   

Our X-ray studies (methods are reported in the supporting information) revealed that the overall protein backbones (tertiary structures) of the native and V143I CA II structures were very similar, with C^α^–C^α^ r.m.s.d. values of less than 0.14 Å (Supplementary Tables S1 and S2). However, careful structural analysis successfully established subtle but clear changes in the active site (CO_2_-binding site, proton-transfer pathway and water-replenishment pathway; EC waters). The key bound water molecules in native and V143I CA II are listed in Supplementary Tables S3 and S4.

### 2.1. CO_2_-binding site around the zinc ion   

**Figure 2:**
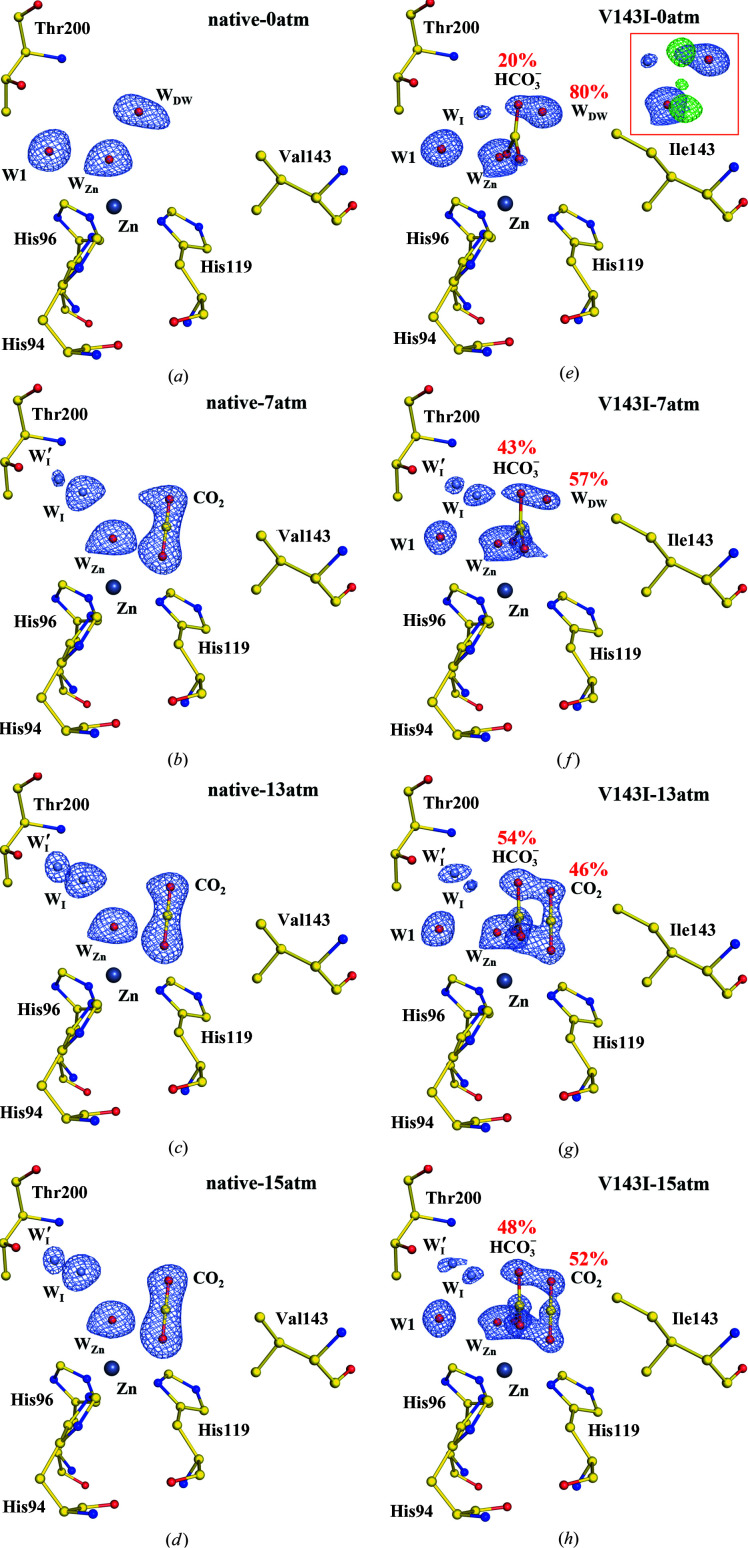
CO_2_/HCO_3_^−^-binding site of native and V143I CA II. The intermediate waters (W_I_ and W_I_′) are coloured steel blue for clarity. The electron density (2*F*_o_ − *F*_c_) is contoured at 1.5σ where not indicated otherwise. (*a*)–(*d*) Native CA II structures. (*e*)–(*h*) V143I CA II structures. W_I_′ at 7, 13 and 15 atm is contoured at 1.25σ and W_I_ at 0 atm is contoured at 1.0σ. Partial occupancies of HCO_3_^−^ and W_DW_ were determined in V143I-0atm and V143I-7atm, and partial occupancies of HCO_3_^−^ and CO_2_ were determined at the higher CO_2_ pressures (see the supporting information). The inset (red box) in V143I-0atm shows the difference map (*F*_o_ − *F*_c_ contoured at 3.0σ; green) when the HCO_3_^−^ molecule is not included in the structure refinement.

Fig. 2 ▸ shows the CO_2_-binding site and crucial water molecules (W_Zn_/W_DW_/W_I_/W_I_′/W1) in the vicinity of the zinc ion. In native CA II [Figs. 2 ▸(*a*)–2 ▸(*d*)], the water molecules (W_Zn_/W_DW_/W1) around the zinc ion are initially well ordered at 0 atm CO_2_ pressure. At higher CO_2_ pressures of 7–15 atm, electron density for the CO_2_ molecule becomes apparent, displacing the deep water (W_DW_). Concurrently, two intermediate waters W_I_ and W_I_′ emerge near Thr200, while W1 disappears at higher CO_2_ pressures. The distance between W_I_ and W2 is ∼4.7 Å, suggesting that the hydrogen-bonded water network that facilitates proton transfer is disrupted when the CO_2_-binding site is fully occupied.

On the other hand, the active site of V143I CA II shows noteworthy modifications. The most striking difference is that HCO_3_^−^ is stabilized and is observable at 0 atm CO_2_ pressure with an estimated occupancy of ∼20% [Fig. 2 ▸(*e*)]. In the absence of experimentally introduced CO_2_, it is likely that the captured HCO_3_^−^ is converted from CO_2_ absorbed into the crystal from ambient air. As the CO_2_ pressure increases, the HCO_3_^−^ occupancy increases to 54% at 13 atm and subsequently decreases slightly to 48% at 15 atm, with increased CO_2_ occupancy [Figs. 2 ▸(*f*)–2 ▸(*h*)]. It is likely that the observed decrease in the HCO_3_^−^ occupancy at 15 atm is owing to steric hindrance from the bound CO_2_ molecule. When superimposed with the previously reported coordinates of HCO_3_^−^ bound to native CA II (PDB entry 2vvb; Sjöblom *et al.*, 2009 ▸), the HCO_3_^−^ position observed in V143I CA II shows noticeable deviations. The HCO_3_^−^ molecule is tilted by 35° with respect to the plane containing W_Zn_, CO_2_ and HCO_3_^−^ in native CA II (Supplementary Fig. S1). In addition, the central C atoms of the two superimposed HCO_3_^−^ molecules in native and V143I CA II are separated by 0.5 Å.

Unlike the HCO_3_^−^ molecule, the CO_2_ molecule is less stable in V143I CA II. For example, CO_2_ shows almost full occupancy at 7 atm in native CA II, while no CO_2_ is visible at 7 atm in V143I CA II, which instead shows the appearance of W_DW_ [Figs. 2 ▸(*b*) and 2 ▸(*f*)]. The CO_2_ molecule appears at higher pressures (13 and 15 atm) with a decreased occupancy of ∼50% [Figs. 2 ▸(*g*) and 2 ▸(*h*)]. The bound CO_2_ is tilted by ∼6° and is situated closer to the zinc ion by 0.34 Å compared with that in native CA II (Supplementary Fig. S1). The distance between the end carbon (C^δ1^) of Ile143 and the CO_2_ molecule is only 3.0–3.2 Å, and this steric disruption seems to affect the critical interactions between the CO_2_ molecule and the hydrophobic pocket, thereby destabilizing it in the active site. It is worth noting that in contrast to native CA II, the bound CO_2_ molecule in V143I CA II is distorted from the plane defined by the bound HCO_3_^−^ molecule, and this seems to be detrimental to the efficient conversion of CO_2_ to HCO_3_^−^.

Finally, the two intermediate waters W_I_ and W_I_′ show different behaviour in V143I CA II. The intermediate water W_I_ is visible even in 0 atm V143I CA II but is not present in 0 atm native CA II [Figs. 2 ▸(*a*) and 2 ▸(*e*)]. However, it is observed that the electron densities of the two intermediate waters were less defined than in native CA II [Figs. 2 ▸(*f*)–2 ▸(*h*)]. These ‘weaker’ intermediate waters were accompanied by the presence of W1 at all pressures (0–15 atm). It is likely that these weak intermediate waters are related to the perturbed structures and dynamical motions of the EC waters, as explained later.

### 2.2. Proton-transfer pathway   

**Figure 3:**
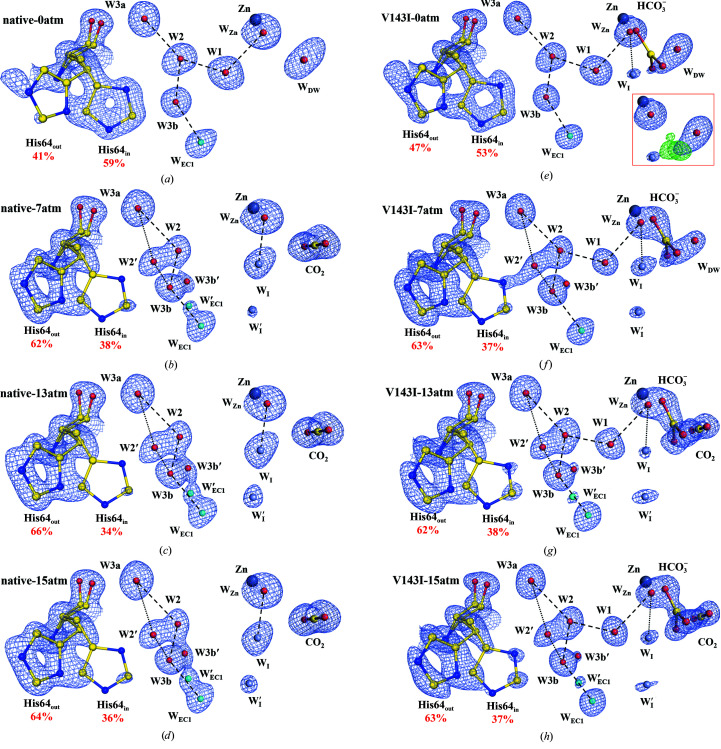
Proton-transfer pathway including the water network and His64. The entrance-conduit waters (W_EC_) are coloured cyan and the intermediate waters (W_I_ and W_I_′) are coloured steel blue for clarity. The electron density (2*F*_o_ − *F*_c_) is contoured at 1.5σ where not indicated otherwise. The major hydrogen bonds between water molecules are represented by dashed lines, while alternative hydrogen bonds that are mutually exclusive are represented by dotted lines. (*a*)–(*d*) Native CA II structures. (*e*)–(*h*) V143I CA II structures. W_I_′ at 7, 13 and 15 atm and W′_EC1_ at 15 and 13 atm are contoured at 1.25σ, and W_I_ at 0 atm is contoured at 1.0σ. The inset (red box) in V143I-0atm shows the difference map (*F*_o_ − *F*_c_ contoured at 3.0σ; green) when the HCO_3_^−^ molecule is not included in the structure refinement.

Fig. 3 ▸ shows the proton-transfer pathway including the water network (W1/W2/W3a/W3b) and His64. In native CA II, the water network is initially well ordered at 0 atm CO_2_ pressure [Fig. 3 ▸(*a*)]. As the CO_2_ pressure increases, W1 disappears and intermediate waters (W_I_ and W_I_′) emerge instead [Figs. 3 ▸(*b*)–3 ▸(*d*)]. The W2 water, which transfers a proton from W1 to His64, shows an alternative position denoted W2′. Considering the steric hindrance between His64_in_ and W2′, it seems that the presence of W2′ pushes His64 towards the ‘out’ conformation (away from the water network). Indeed, His64 shows a net movement from the ‘in’ to the ‘out’ conformation as W2′ becomes prominent at higher CO_2_ pressures. W3a shows little change, but W3b shows an alternative water position, W3b′, and is found to interact with one of the entrance waters W_EC1_ (and its alternative position W′_EC1_).

Similarly, in V143I CA II W2 shows the same alternative position W2′, and His64 shows the same ‘in’ to ‘out’ flip, with similar occupancies as observed in native CA II with increasing CO_2_ pressure [Figs. 3 ▸(*e*)–3 ▸(*h*)]. W3b and W_EC1_ also show the same alternative positions, although their electron densities are slightly weaker. As the distance between W2 and the N atom (N^δ1^) of His64_in_ is relatively long (3.3 Å), efficient proton transfer seems to depend on the dynamical motions of W2/W2′ and His64_in_/His64_out_. These motions are quite similar in native and V143I CA II and therefore proton transfer is not significantly impacted in the variant.

### 2.3. Water-replenishment pathway   

**Figure 4:**
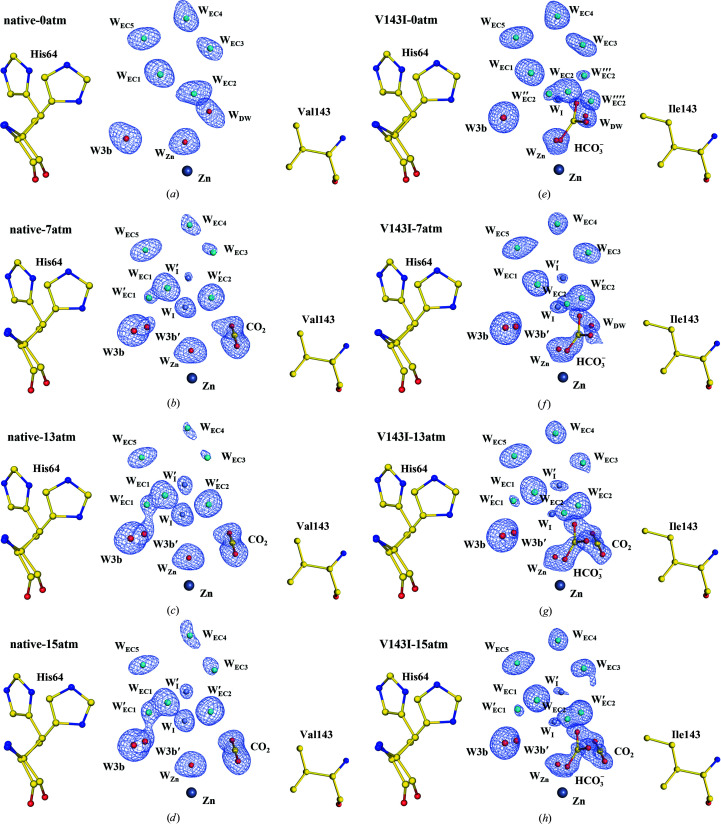
The water-replenishment pathway including entrance-conduit waters (cyan) and intermediate waters (steel blue). The electron density (2*F*_o_ − *F*_c_) is contoured at 1.5σ where not indicated otherwise. (*a*)–(*d*) Native CA II structures. W_EC3_ at 7 and 13 atm and W_EC4_ at 15 atm are contoured at 1.25σ, and W_EC3_ at 15 atm is contoured at 1.0σ. (*e*)–(*h*) V143I CA II structures. W_I_′ at 7, 13 and 15 atm and W′_EC1_ at 15 and 13 atm are contoured at 1.25σ and W_I_ at 0 atm is contoured at 1.0σ. Compared with native CA II, V143I CA II shows significantly perturbed positions for W_EC2_.

Fig. 4 ▸ shows the water-replenishment pathway consisting of the ordered EC waters (W_EC1_/W_EC2_/W_EC3_/W_EC4_/W_EC5_). In native CA II, the five W_EC_ waters are well ordered at 0 atm CO_2_ pressure [Fig. 4 ▸(*a*)]. As the CO_2_ pressure increases, W_EC1_ shows an alternative position W′_EC1_, and W_EC2_ shifts to an alternative position W′_EC2_ [Figs. 4 ▸(*b*)–4 ▸(*d*)]. These dynamical motions of W_EC1_ and W_EC2_ are accompanied by the emergence of the intermediate waters W_I_ and W_I_′. The two intermediate waters are located deep within the entrance conduit near the active site and are transiently stabilized via hydrogen bonding to several W_EC_ waters (W_EC2_, W_EC3_ and W_EC5_ and their alternative positions). The short distance (2.2 Å) between W_I_ and W_I_′ suggests that W_I_′ can rapidly shift to the W_I_ position as W_I_ refills the vacant water positions (W1/W_Zn_/W_DW_) during catalysis. Previous studies suggest that the intermediate waters (W_I_ and W_I_′) play a critical role in the rapid replenishment of the active-site water network during catalysis and therefore could influence the overall catalytic rate (Kim *et al.*, 2018 ▸).

Among the five W_EC_ waters in V143I CA II, W_EC2_ is located close to the mutated residue Ile143. Indeed, the W_EC1_ to W_EC4_ waters show similar structures and dynamical motions as in native CA II, but W_EC2_ shows a significantly distinct behaviour. Initially, at 0 atm CO_2_ pressure, the W_EC2_ water shows multiple alternative positions (W′′_EC2_, W′′′_EC2_ and W′′′′_EC2_) [Fig. 4 ▸(*e*)]. As the CO_2_ pressure increases, these alternative positions disappear and both the W_EC2_ and W′_EC2_ waters are observed instead [Figs. 4 ▸(*f*)–4 ▸(*h*)]. Compared with native CA II, W′′_EC2_, W′′′_EC2_ and W′′′′_EC2_ are new alternative positions that are observed only at 0 atm CO_2_ pressure in V143I CA II. Along with the perturbed dynamical motions of the W_EC2_ water, much weakened electron densities of the intermediate waters W_I_ and W_I_′ are observed. It should be noted that among the five EC waters, W_EC2_ is unique in that it interacts with all three of the key waters, W1, W_I_ and W_I_′. Therefore, the fact that the structures and dynamical motions of the W_EC2_ waters are significantly perturbed in V143I CA II is likely to account for the stabilization of W1 but the destabilization of the two intermediate waters.

Another interesting aspect of W_EC2_ is that its alternative position W′′′′_EC2_ is situated close to the bound HCO_3_^−^. The distances between W′′′′_EC2_ and the closest O atom and the C atom of HCO_3_^−^ are 1.4 and 2.5 Å, respectively (Supplementary Table S5). Since W′′′′_EC2_ is too close to the bound HCO_3_^−^, W′′′′_EC2_ and HCO_3_^−^ cannot coexist in tandem. Indeed, W′′′′_EC2_ is only visible in V143I CA II at 0 atm, when the HCO_3_^−^ occupancy is low, and disappears as the HCO_3_^−^ occupancy increases at higher CO_2_ pressures [Figs. 4 ▸(*e*)–4 ▸(*h*)]. It seems that the relationship between W′′′′_EC2_ and HCO_3_^−^ is analogous to the relationship between W_DW_ and CO_2_. In native CA II, the hydrophobic cavity produces an electrostatic environment in which the deep water (W_DW_) can be locally stabilized around the zinc ion and then replaced with one of the O atoms of CO_2_ upon CO_2_ binding. In V143I CA II, the altered hydrophobic cavity owing to the V143I mutation produces a slightly different electrostatic environment in which an additional water position (W′′′′_EC2_) can be locally stabilized around the zinc ion and subsequently replaced with one of the O atoms in HCO_3_^−^ during CA II catalysis. The release of the W′′′′_EC2_ molecule upon HCO_3_^−^ binding seems to reduce the entropic cost of the process, analogous to the release of W_DW_ upon CO_2_ binding in native CA II. Thus, it is likely that the altered multiple conformations of W_EC2_ allow HCO_3_^−^ to bind more easily and firmly in a tilted configuration at the active site.

## 3. Discussion   

In this study, we have successfully identified the ‘fine’ structural changes in the CA II intermediates induced by a single-residue mutation at the active site. The V143I mutation in CA II produces steric hindrance and induces subtle changes in the electrostatic environment of the active site. The resulting effects on the CA II intermediates can be summarized as follows: (i) the dynamical motions and the allowed configurations of CO_2_ are slightly restricted and the binding affinity of HCO_3_^−^ is increased with a distorted configuration and (ii) the EC water network in the water-replenishment pathway is restructured, while (iii) the proton-transfer dynamics are mostly unaffected. These detailed structural insights can now be used to assess the modifications in the reaction rate constants (*k*_1_, *k*_−1_, *k*_2_ and *k*_3_) and the corresponding free-energy profiles during the CO_2_-hydration reaction of CA II.

**Figure 5:**
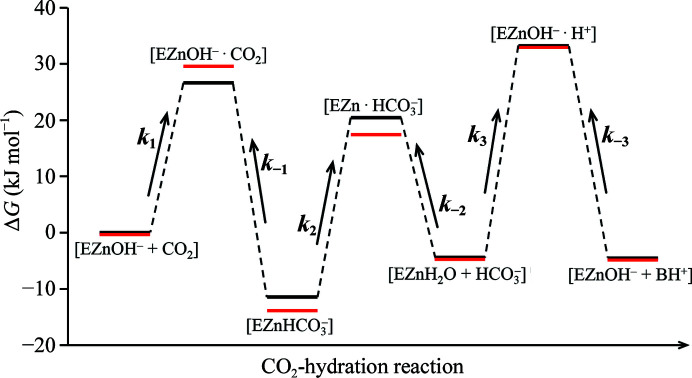
Estimated free-energy profiles for the CO_2_-hydration reaction catalyzed by CA II. The energy states of native CA II (black) are from a previous study (Behravan *et al.*, 1990 ▸). The energy states of V143I CA II (red) are qualitatively estimated with respect to the native form by considering the structural information and the variations in the reaction rate constants. Note that the energy level of [EZnH_2_O + HCO_3_^−^] in the V143I variant is assumed to be the same as that in native CA II. The depicted energy gaps are not to scale.

Firstly, the V143I mutation restricts the configurational freedom of the CO_2_ molecule within the hydrophobic pocket of CA II. Previous mutational studies on hydrophobic pocket residues (Val121 and Val143) suggest that the hydrophobic pocket of native CA II is involved in ‘ushering’ the CO_2_ molecule to the zinc hydroxide in the active site, but does not directly interact with the CO_2_ molecule to hold it in a specific orientation. Therefore, it is most likely that the CO_2_ molecule in aqueous solution is guided to the hydrophobic pocket of CA II but retains some degrees of freedom with regard to the acceptable configurations (positions and orientations) around the zinc hydroxide that eventually facilitate its rapid conversion into HCO_3_^−^. In the V143I variant, although the hydrophobicity of the active-site pocket is increased, the added methyl group appears to sterically restrict the number of favourable configurations and the dynamical motions accessible to the CO_2_ molecule within the cavity. This estimation is supported by the crystallographic observation that the CO_2_ molecule is distorted towards the zinc ion, tilted by ∼6° and is destabilized with lower occupancies in the V143I variant. Consequently, interconversion from CO_2_ to HCO_3_^−^ becomes less efficient, leading to a reduced *k*_1_ (*k*_1_^V143I^ < *k*_1_^native^). The lower *k*_1_ value also implies an enhanced activation-energy barrier for the step [Arrhenius relationship: *k*_1_(*T*) = *A*exp(−*E*_1_/*RT*), where *A* is a pre-exponential constant, *R* is the molar gas constant, *T* is the absolute temperature and *E*_1_ is the activation energy] (Fig. 5 ▸).

Secondly, the V143I mutation induces a slightly different electrostatic environment in the hydrophobic cavity, thereby altering the location and dynamics of the W_EC2_ water. It seems that one of these altered W_EC2_ waters (W′′′′_EC2_) increases the binding affinity of the HCO_3_^−^ molecule in the active site. This stronger binding of HCO_3_^−^ suggests that the free energy of the enzyme–product (EZnHCO_3_^−^) complex is lowered in the V143I variant (Fig. 5 ▸). In conjunction with the larger activation energy for the *k*_1_ reaction, it can be deduced that the activation energy for the reverse reaction *k*_−1_ is increased even further, leading to a reduced *k*_−1_ value (*k*_−1_^V143I^ < *k*_−1_^native^). On the other hand, the alterations of the W_EC2_ water in the V143I variant make the intermediate waters (W_I_ and W_I_′) slightly less stable, therefore possibly slowing down the replenishment of the active-site water network and HCO_3_^−^ dissociation. This results in a reduced *k*_2_ value (*k*_2_^V143I^ < *k*_2_^native^).

Thirdly, the V143I mutation seems to have little effect on the kinetics of the proton-transfer reaction. It is observed that W1 is more stabilized in the V143I variant intermediates. It should be noted that the intermolecular proton transfer can occur via the fully established water network W_Zn_→W1→W2→His64. This implies that if a mutation induces the destabilization of W1, proton transfer could be significantly perturbed. However, stabilization of W1 as in the V143I variant does not necessarily suggest a faster proton-transfer process. Rather, it is the dynamical motions of W2 and His64 that have a more critical influence on the proton-transfer rate. Our results suggest that both W2 and His64 show very similar dynamical motions. Taken together, it seems that the overall proton-transfer rate *k*_3_ is not significantly altered in the V143I mutant (*k*_3_^V143I^ ≃ *k*_3_^native^).

The interplay between the modified reaction rate constants, as discussed above, now allows us to determine the effect of the V143I mutation on the measured kinetic parameters (*k*_cat_ and *k*_cat_/*K*_m_; Nair *et al.*, 1991 ▸; Krebs, Ippolito *et al.*, 1993 ▸). The steady-state kinetic parameters for the CO_2_-hydration reaction are listed in Table 1 ▸. The *k*_cat_ value shows little change, but the second-order rate constant *k*_cat_/*K*_m_ shows an approximately tenfold decrease in the V143I variant. The parameter *k*_cat_ contains rate constants from the initial enzyme–substrate complex through the remaining steps, including proton transfer. Therefore, for the proposed mechanistic scheme (equations 1 and 2), *k*_cat_ can effectively be represented as *k*_cat_ = *k*_2_ *k*_3_/(*k*_2_ + *k*_3_). On the other hand, the ratio *k*_cat_/*K*_m_ contains rate constants for the initial association of the substrate CO_2_ through the dissociation of the product HCO_3_^−^. Hence, *k*_cat_/*K*_m_ only contains rate constants from equation 1 (and not equation 2) and is represented as *k*_cat_/*K*_m_ = *k*_1_ *k*_2_/(*k*_−1_ + *k*_2_).

It is known that in native CA II the HCO_3_^−^-dissociation process (*k*_2_) is much faster than the reverse interconversion from HCO_3_^−^ to CO_2_ (*k*_−1_) and the proton-transfer rate (*k*_3_), *i.e.* *k*_2_^native^ ≫ *k*_−1_^native^, *k*_2_^native^ ≫ *k*_3_^native^, with *k*_2_^native^ ≃ 10*k*_−1_^native^, *k*_2_^native^ ≃ 15*k*_3_^native^ and *k*_−1_^native^ ≃ 1.5*k*_3_^native^ (Table 1 ▸). Combining this information with the insights gleaned from our study (*k*_1_^V143I^ < *k*_1_^native^, *k*_−1_^V143I^ < *k*_−1_^native^, *k*_2_^V143I^ < *k*_2_^native^ and *k*_3_^V143I^ ≃ *k*_3_^native^), we can estimate the effect of the V143I point mutation on the observed kinetic parameter *k*_cat_ in the following way. In the native state [*k*_cat_]^native^ = *k*_2_^native^ *k*_3_^native^/(*k*_2_^native^ + *k*_3_^native^) ≃ *k*_3_^native^ (using *k*_2_^native^ ≫ *k*_3_^native^), indicating that the turnover rate in the native is almost the same as the proton-transfer rate and that the proton-transfer process is the rate-limiting step. In the V143I variant [*k*_cat_]^V143I^ = *k*_2_^V143I^ *k*_3_^V143I^/(*k*_2_^V143I^ + *k*_3_^V143I^) ≃ *k*_3_^native^[*k*_2_^V143I^/(*k*_2_^V143I^ + *k*_3_^V143I^)] (using *k*_3_^V143I^ ≃ *k*_3_^native^), and the experimental measurement (Table 1 ▸) suggests that [*k*_cat_]^V143I^ ≃ [*k*_cat_]^native^ ≃ *k*_3_^native^, implying that [*k*_2_^V143I^/(*k*_2_^V143I^ + *k*_3_^V143I^)] ≃ 1, or *k*_2_^V143I^ ≫ *k*_3_^V143I^. This result indicates that HCO_3_^−^ dissociation (*k*_2_) remains faster than proton transfer (*k*_3_) in the V143I variant and that the proton-transfer process is still the rate-limiting step in the V143I variant. The negligible reduction in *k*_2_^V143I^ suggests that its activation energy is not significantly increased (Fig. 5 ▸).

The second-order rate constant *k*_cat_/*K*_m_ shows the following relationship in the native state: [*k*_cat_/*K*_m_]^native^ = *k*_1_^native^ *k*_2_^native^/(*k*_−1_^native^ + *k*_2_^native^) ≃ *k*_1_^native^ (using *k*_2_^native^ ≫ *k*_−1_^native^). This indicates that [*k*_cat_/*K*_m_]^native^ mostly reflects the CO_2_-binding step and its interconversion to HCO_3_^−^. On the other hand, in the V143I variant both the dissociation of HCO_3_^−^ (*k*_2_) and the reverse interconversion (*k*_−1_) from HCO_3_^−^ to CO_2_ are retarded (*k*_2_^V143I^ < *k*_2_^native^ and *k*_−1_^V143I^ < *k*_−1_^native^), leaving the relation *k*_2_^V143I^ ≫ *k*_−1_^V143I^ unresolved. However, the relation *k*_2_^V143I^ ≫ *k*_3_^V143I^ ≃ *k*_3_^native^ obtained from the *k*_cat_ analysis above suggests that *k*_2_^V143I^ is still an order of magnitude larger than *k*_3_^native^, while *k*_−1_^V143I^ < *k*_−1_^native^ ≃ 1.5*k*_3_^native^ suggests that *k*_−1_^V143I^ is comparable to or less than *k*_3_^native^, thereby ensuring that the relation *k*_2_^V143I^ ≫ *k*_−1_^V143I^ remains valid. Thus, we can estimate the *k*_cat_/*K*_m_ in the V143I variant in the following manner: [*k*_cat_/*K*_m_]^V143I^ = *k*_1_^V143I^ *k*_2_^V143I^/(*k*_−1_^V143I^ + *k*_2_^V143I^) ≃ *k*_1_^V143I^ (using *k*_2_^V143I^ ≫ *k*_−1_^V143I^). This estimation indicates that the reduced [*k*_cat_/*K*_m_]^V143I^ is mostly owing to the decrease in *k*_1_, reflecting the slower CO_2_ binding and interconversion to HCO_3_^−^ in the V143I variant. It should be noted that the reduction in the *k*_−1_ value has little effect on [*k*_cat_/*K*_m_]^V143I^ in the direction of CO_2_ hydration, as long as the reduction in *k*_2_ is small enough to keep the relation *k*_2_^V143I^ ≫ *k*_−1_^V143I^ valid. However, it is expected that the reduction in the *k*_−1_ value would have significant consequences for the HCO_3_^−^-dehydration direction.

Finally, considering that *k*_cat_ ≃ *k*_3_ and [*k*_cat_/*K*_m_] ≃ *k*_1_ in both native and V143I CA II, the Michaelis constant *K*_m_ is expressed in the following way: *K*_m_^native^ = *k*_3_^native^/*k*_1_^native^ and *K*_m_^V143I^ = *k*_3_^V143I^/*k*_1_^V143I^. Consequently, *K*_m_^V143I^ > *K*_m_^native^ can be obtained using the estimated relationships *k*_1_^V143I^ < *k*_1_^native^ and *k*_3_^V143I^ ≃ *k*_3_^native^. The relationship shows that the substrate concentration needed to reach half of the maximum reaction velocity is larger in V143I CA II mainly owing to the slower CO_2_ binding and interconversion to HCO_3_^−^.

Although our study was performed for a single point mutation within the hydrophobic pocket (V143I), our approach and interpretations can be extended to arbitrary mutations in CA II. Considering the forward CO_2_-hydration direction, the mutation can first perturb substrate funnelling into the hydrophobic pocket via steric hindrance, thereby limiting the configurations that allow its efficient conversion into product. This influence is directly reflected in *k*_cat_/*K*_m_, but not in *k*_cat_. Secondly, the mutation can structurally distort the proton-transfer pathway by perturbing the water network or its associated stabilizing residues, and this effect is directly reflected in *k*_cat_ but not in *k*_cat_/*K*_m_. Thirdly, the mutation can alter the product-dissociation process via direct steric hindrance or perturbations in the water-replenishment pathway. This influence can be intricate and is reflected both in *k*_cat_ and *k*_cat_/*K*_m_. On the other hand, the mutation can affect the reverse reaction, the interconversion from product to substrate and substrate dissociation, but this has little influence on either *k*_cat_ or *k*_cat_/*K*_m_ in the hydration direction, as long as the reverse interconversion process is much slower than the product-dissociation process.

## 4. Conclusion   

We systematically studied the effect of a single-residue mutation on the CA II catalytic pathway at atomic resolution. We have successfully captured the high-resolution intermediate states of the V143I variant and shown clearly that the single point mutation induces noticeable changes in substrate and product binding at the active site and in the water-replenishment pathway, but has little effect on the proton-transfer pathway. The structural information was then utilized to estimate the reaction rate constants and the free-energy profiles during the catalytic cycle, unravelling the effect of the point mutation on the altered kinetic parameters. We believe that the detailed and systematic approach in our CA II study can be extended to identify the specific roles of target amino-acid residues in many other biologically important enzymes. We also anticipate that our detailed descriptions could serve as a reference point for future theoretical and computational studies that may lead to an advanced understanding of enzyme mechanisms at the quantum-chemistry level.

## 5. Related literature   

The following references are cited in the supporting information for this article: Emsley *et al.* (2010 ▸), Forsman *et al.* (1988 ▸), Henderson (1990 ▸), Khalifah *et al.* (1977 ▸), Kim *et al.* (2005 ▸, 2006 ▸, 2013 ▸, 2016 ▸), McPherson (1982 ▸), Murshudov *et al.* (2011 ▸), Otwinowski & Minor (1997 ▸) and Winn *et al.* (2011 ▸).

## Supplementary Material

PDB reference: human carbonic anhydrase II, native, 0 atm CO_2_, 6km3

PDB reference: 7 atm CO_2_, 6km4

PDB reference: 13 atm CO_2_, 6km5

PDB reference: 15 atm CO_2_, 6km6

PDB reference: V143I variant, 0 atm CO_2_, 6klz

PDB reference: 7 atm CO_2_, 6km0

PDB reference: 13 atm CO_2_, 6km1

PDB reference: 15 atm CO_2_, 6km2

Supplementary Methods, Tables and Figures. DOI: 10.1107/S2052252520011008/jt5048sup1.pdf

## Figures and Tables

**Table 1:** Steady-state kinetic parameters for CO_2_ hydration by native and V143I CA II

	*k*_cat_/*K*_m_ (µ*M* ^−1^ s^−1^)	*k*_cat_ (µs^−1^)	*K*_m_ (m*M*)	*k*_1_ † (*M* ^−1^ s^−1^)	*k*_−1_ † (s^−1^)	*k*_2_ † (s^−1^)	*k*_−2_ † (*M* ^−1^ s^−1^)	*k*_3_ † (s^−1^)
Native	89 ± 7‡/120§	0.93 ± 0.05‡/1.0§	11 ± 1‡	1.3 × 10^8^	1.8 × 10^6^	1.7 × 10^7^	2.0 × 10^8^	1.2 × 10^6^
V143I	11 ± 1‡/9.3 ± 0.3§	1.0 ± 0.2‡/0.7 ± 0.1§	100 ± 24‡	—	—	—	—	—

† From Behravan *et al.* (1990 ▸).

‡ From Fierke *et al.* (1991 ▸).

§ From West *et al.* (2012 ▸).
