# Supplementary material for: Structural insights into the effect of active-site mutation on the catalytic mechanism of carbonic anhydrase
Source: IUCrJ. 2020 Sep 9;7(Pt 6):985–94. doi: 10.1107/S2052252520011008 (PMC7642793; doi:10.1107/S2052252520011008)
Supplement: Supplementary file 1 [file m-07-00985-sup1.pdf]

IUCrJ

Volume 7 (2020)

Supporting information for article:

Structural insights into the effect of active-site mutation on the catalytic mechanism of carbonic anhydrase

Jin Kyun Kim, Cheol Lee, Seon Woo Lim, Jacob Andring, Adhikari Aniruddha, Robert McKenna and Chae Un Kim

S1. Protein expression and purification

The V143I CA II variant was made by site-directed mutagenesis using an expression vector with an CA II coding region. The single-point mutation was made using the QuikChange II and QuikChange Lightning kits from Agilent. Verification of mutation was accomplished by DNA sequencing of the entire CA II coding region, followed by simulated translation using *ExPASY* translate. The expression of variant involved transformation of the mutated plasmid into *Escherichia coli* BL21(DE3)pLysS cells, a cell line specific for protein expression and one that does not express endogenous CA under our experimental conditions. The cells were transformed and expressed at 37 °C in LB, followed by induction with 1 mM isopropyl thiogalactoside when the bacterial growth reached an OD_{600} of 0.6. 1 mM zinc sulfate was also added to provide a source of zinc for the proper folding and functioning of the enzyme. Cells were harvested 4 h after induction, spun down, and stored in a −80 °C freezer overnight. The cell pellets were lysed with lysozyme and homegenation, and the lysate was purified via affinity chromatography using p-(aminomethyl) benzenesulfonamide resin. (Khalifah *et al.*, 1977) CA II was eluted off the column by the addition of azide, and azide was subsequently removed via buffer exchange. Following purification, the enzyme concentration was determined via UV spectrometry, monitored at 280 nm followed by an SDS page gel for purity. Native CA II was expressed in a recombinant strain of *Escherichia coli* [BL21 (DE3) pLysS] containing a plasmid encoding the CA II gene. (Forsman *et al.*, 1988) Purification was performed following the same protocol as above.

S2. Crystallization

Crystals of native and V143I CA II were obtained using the hanging drop vapor diffusion method. (McPherson, 1982) A 6 µl drop of equal volumes of protein (3 µl) and the well-solution (3 µl) was equilibrated against 500 µl of the well-solution (1.3-1.4 M sodium citrate, 100 mM Tris·HCl pH 7.8-8.0) at RT (~20 °C). (Fisher *et al.*, 2007) Crystals grew to an approximate size of ~ 30 × 100 × 100 μm^3 within a few days.

S3. CO_2 entrapment

Cryo-trapping the intermediate states of CA II was previously achieved by cryocooling CA II crystals under CO_2 pressure, (Kim *et al.*, 2005, Kim *et al.*, 2013) leading to the capture of CO_2 in the active site of CA II for the first time. (Domsic *et al.*, 2008) More recently, series of intermediate states have been tracked in CA II by controlling the internal CO_2 pressure levels. (Kim *et al.*, 2016, Kim *et al.*, 2018) In this study, the CO_2 entrapment was carried out using a high-pressure cryo-cooler for X-ray crystallography (HPC-201, Advanced Design Consulting, USA). The native and V143I CA II crystals were first soaked in a cryo-solution containing 20% (*v*/*v*) glycerol supplemented to the reservoir solution. The crystals were then coated with mineral oil to prevent dehydration, and loaded into the base of high-pressure tubes. (Kim *et al.*, 2005) The coated mineral oil worked as a CO_2 buffering medium as well, aiding in the absorption of CO_2 into the crystals. (Kim *et al.*, 2006) The crystals were

pressurized in the pressure tubes with CO_2 gas (0 atm (no pressurization), 7 atm, 13 atm, and 15 atm) at room temperature. After a wait of about 5 minutes, the crystals were cryocooled in liquid nitrogen (77 K) under sustained CO_2 pressure. Once the CO_2 bound crystals were fully cryocooled, the CO_2 gas pressure was withdrawn, and the crystal samples were stored in a liquid nitrogen dewar for subsequent X-ray data collection.

S4. X-ray diffraction and data collection

Diffraction data of native and V143I CAII were collected at Pohang Light Source II (wavelength 0.8856 Å, beam size 100 μm, crystal-to-detector distance 113.80 mm, at 100 K) and at the Cornell High Energy Synchrotron Source (wavelength 0.9179 Å, beam size 100 μm, crystal-to-detector distance 100 mm, at 100 K), respectively. Data were collected using the oscillation method in intervals of 1° step on an ADSC Quantum 270 CCD detector (Area Detector Systems Corporation, USA). A total of 360 images were collected on each of the CA II crystal data sets.

For each data set, a fresh pressure-cryocooled crystal was used. The absorbed X-ray dose for a single data set was less than ~ $5x10^5$ Gy for both the native and the V143I CA II, which is much less than the Henderson dose limit of ~ $1.0 x10^7$ Gy. (Henderson, 1990) Moreover, it was previously verified that X-ray radiation dose of up to 10^7 Gy does not induce apparent changes in the enzyme active site. (Kim *et al.*, 2016) This ensured that the active site rearrangements described in our present study remained unaffected by the incident X-ray radiation. Indexing, integration, and scaling were performed using HKL2000. (Otwinowski & Minor, 1997) The data processing statistics are given in Tables S1 & S2.

S5. Structure determination and model refinement

The structures of native and Va143 CA II pressure series were determined using the CCP4 program suite. (Winn et al., 2011) Prior to refinement, a random 5% of the data were flagged for R_{free} analysis. The crystal structures (PDB codes of 5YUK and 3U7C for the native and the V143I CA II, respectively) were used as the initial phasing models. (West *et al.*, 2012, Kim *et al.*, 2018) The maximum likelihood refinement (MLH) was carried out using REFMAC5. (Murshudov *et al.*, 2011) The refined structures were manually checked using the molecular graphics program COOT. (Emsley & Cowtan, 2010) Reiterations of maximum likelihood refinement (MHL) were carried out with anisotropic B factor.

On completion of the structural refinements as described above, systematic refinements were further carried out to accurately determine the partial occupancies of the HCO_3^-/ (W_{DW} & W_{Zn}), HCO_3^- /(CO_2&W_{Zn}), and $His64_{in}$/$His64_{out}$ configurations (primary/secondary configurations). A total of 99 structures were prepared for each of the native and V143I CA II pressure series, in which the occupancies of the first and the second configurations were changed in incremental steps of 1 % (i.e., the 1st structure with 1% in the first configuration and 99% in the second configuration, the 2nd structure with 2% in the first and 98% in the second, …, the 99th structure with 99% in the first and 1% in the

second). MLH refinements were carried out in parallel for all the 99 structures. After MLH refinements, the overall R-factor as a function of partial occupancy of the first configuration was obtained, and it was fitted into a quadratic function (Fig. S2&S3). The partial occupancy values of the first configuration were determined such that they minimized the overall R-factor. Details on the refinement statistics are given in Tables S1 & S2. All structural figures were rendered with PyMol (Schrödinger, LLC).

S6. Structural analysis of the bound water molecules

To compare the bound water molecules in the active site and the entrance conduit, we carefully refined water molecules based on the PDB and COOT validation checks and the electron density maps (cutoff level of 1σ in $2F_o$-F_c electron density map). We have tested the consistency and reproducibility of the bound water molecules in the active site and the entrance conduit carefully. There are several closely positioned water molecules in the active site and the entrance conduit of the CA II structures. Since most of these waters exist transiently, it is allowed that they can be located closer than the normal stably bound water molecules. In this regard, water molecules closely located near the active site and entrance conduit regions were not excluded in the final coordinates. The important bound water molecules addressed in the main manuscript are listed in Tables S3 and S4. The distance information between CO_2, HCO_3^-, Val143, Ile143, and important water molecules is listed in Table S5.

Table S1 Data collection and refinement statistics for the native CA II.

Values in parentheses are for the highest-resolution shell.

CO_2 pressure (PDB code)	0 atm (6KM3)	7 atm (6KM4)	13 atm (6KM5)	15 atm (6KM6)
**Data collection**				
Space group	$P2_1$	$P2_1$	$P2_1$	$P2_1$
Cell dimensions				
a, *b*, *c* (Å)	42.14, 41.20, 72.11	42.18, 41.32, 72.23	42.17, 41.32, 71.99	42.15, 41.31, 71.96
β(°)	104.17	104.04	104.07	104.09
Resolution (Å)	30-1.15 (1.17-1.15)	30-1.15 (1.17-1.15)	30-1.15 (1.17-1.15)	30-1.15 (1.17-1.15)
R_{sym} (%)	7.0 (19.5)	5.4 (39.5)	4.9 (43.6)	5.4 (76.9)
$I/\sigma(I)$	24.7 (11.9)	26.0 (5.5)	33.1 (4.8)	31.6 (2.2)
Completeness (%)	94.6 (91.9)	97.9 (96.5)	99.4 (98.5)	95.9 (93.2)
Redundancy	7.0 (7.3)	6.6 (6.7)	6.6 (6.7)	6.0 (5.3)
**Refinement**				
Resolution (Å)	1.15	1.15	1.15	1.15
No. reflections	80,768	84,072	84,726	81,959
R_{work} / R_{free} (%)	10.36 / 12.78	9.80 / 12.42	10.28 / 12.88	10.52 / 13.36
No. atoms				
Protein	2,106	2,118	2,107	2,107
Ligand/ion	1 glycerol	2 CO_2, 1 glycerol	2 CO_2, 1 glycerol	2 CO_2, 1 glycerol
Water	271	383	383	383
B-factors				
Protein (main / side chain)	9.93 / 13.64	10.57 / 14.61	12.06 / 16.10	13.45 / 17.63
Ligand/ion	19.49 (glycerol)	16.00 (first CO_2), 28.16 (second CO_2), 18.50 (glycerol)	15.80 (first CO_2), 29.36 (second CO_2), 20.52 (glycerol)	16.18 (first CO_2), 32.00 (second CO_2), 22.94 (glycerol)
Water	24.28	30.07	31.72	33.82
R.m.s. deviations				
Bond lengths (Å)	0.031	0.029	0.030	0.029
Bond angles (°)	2.456	2.368	2.455	2.367
Cα r.m.s.d. from 0 atm structure (Å)	–	0.1219	0.1269	0.1263

Table S2 Data collection and refinement statistics for the V143I CA II.

Values in parentheses are for the highest-resolution shell.

CO_2 pressure (PDB code)	0 atm (6KLZ)	7 atm (6KM0)	13 atm (6KM1)	15 atm (6KM2)
**Data collection**				
Space group	$P2_1$	$P2_1$	$P2_1$	$P2_1$
Cell dimensions				
a, *b*, *c* (Å)	42.21, 41.32, 72.18	42.30, 41.50, 72.14	42.29, 41.45, 72.08	42.30, 41.46, 72.08
β(°)	104.39	104.23	104.18	104.16
Resolution (Å)	30-0.90 (0.92-0.90)	30-0.93 (0.95-0.93)	30-1.05 (1.07-1.05)	30-0.90 (0.92-0.90)
R_{sym} (%)	7.9 (45.6)	8.4 (50.4)	6.8 (45.4)	8.3 (65.3)
$I/\sigma(I)$	22.6 (2.6)	20.4 (2.2)	32.4 (4.8)	20.6 (1.8)
Completeness (%)	90.0 (68.8)	93.7 (77.5)	94.1 (90.7)	95.7 (80.7)
Redundancy	5.8 (3.3)	5.6 (3.2)	7.7 (7.8)	5.6 (3.0)
**Refinement**				
Resolution (Å)	0.90	0.93	1.05	0.90
No. reflections	160,206	152,358	106,239	171,296
R_{work} / R_{free} (%)	10.81 / 12.14	10.77 / 12.19	10.35 / 12.14	10.99 / 12.22
No. atoms				
Protein	2,285	2,294	2,285	2,285
Ligand/ion	1 HCO_3^-, 1 glycerol	1 HCO_3^-, 1 CO_2, 1 glycerol	1 HCO_3^-, 2 CO_2, 1 glycerol	1 HCO_3^-, 2 CO_2, 1 glycerol
Water	388	390	394	390
B-factors				
Protein (main / side chain)	8.78 / 11.60	9.08 / 11.99	8.71 / 11.44	7.79 / 10.44
Ligand/ion	6.29 (HCO_3^-), 14.77 (glycerol)	11.03 (HCO_3^-), 17.55 (second CO_2), 13.13 (glycerol)	8.29 (HCO_3^-) 9.26 (first CO_2), 13.55 (second CO_2), 13.27 (glycerol)	6.86 (HCO_3^-) 9.23 (first CO_2), 12.07 (second CO_2), 12.03 (glycerol)
Water	24.83	24.43	23.80	21.97
R.m.s. deviations				
Bond lengths (Å)	0.034	0.030	0.029	0.030
Bond angles (°)	2.763	2.582	2.574	2.568
Cα r.m.s.d. from 0 atm structure (Å)	–	0.1159	0.1320	0.1341
Cα r.m.s.d. from native structure (Å)	0.1243	0.1291	0.1126	0.1157

Table S3 List of key bound water molecules in the native CA II.

CO_2 pressure (PDB code)	0 atm (6KM3)	7 atm (6KM4)	13 atm (6KM5)	15 atm (6KM6)
W_{DW}	A 589	–	–	–
W_{Zn}	A 464	A 431	A 447	A 432
W_I	–	A 482	A 515	A 498
W_I'	–	A 638	A 636	A 627
W1	A 473	–	–	–
W2	A 623	A 682	A 676	A 673
W2′	–	A 401	A 401	A 401
W3a	A 530	A 544	A 544	A 525
W3b	A 458	A 451	A 464	A 477
W3b′	–	A 700	A 697	A 697
W_{EC1}	A 624	A 672	A 665	A 664
W_{EC1}'	–	A 705	A 702	A 705
W_{EC2}	A 609	–	–	–
W_{EC2}'	–	A 684	A 688	A 685
W_{EC3}	A 639	A 715	A 726	A 722
W_{EC3}'	–	–	–	–
W_{EC3}''	–	–	–	–
W_{EC4}	A 659	A 755	A 753	A 754
W_{EC5}	A 444	A 432	A 432	A 422

Table S4 List of key bound water molecules in the V143I CA II.

CO_2 pressure (PDB code)	0 atm (6KLZ)	7 atm (6KM0)	13 atm (6KM1)	15 atm (6KM2)
W_{DW}	A 401	A 402	–	–
W_{Zn}	A 412	A 401	A 401	A 401
W_I	A 403	A 403	A 402	A 402
W_I'	–	A 665	A 661	A 662
W1	A 514	A 545	A 579	A 586
W2	A 676	A 681	A 680	A 679
W2′	–	A 404	A 403	A 404
W3a	A 564	A 546	A 556	A 551
W3b	A 484	A 450	A 449	A 458
W3b′	–	A 685	A 687	A 683
W_{EC1}	A 672	A 673	A 670	A 666
W_{EC1}'	–	A 682	A 691	A 688
W_{EC2}	A 631	A 662	A 650	A 652
W_{EC2}'	–	A 424	A 425	A 429
W_{EC2}''	A 661	–	–	–
W_{EC2}'''	A 657	–	–	–
W_{EC2}''''	A 402	–	–	–
W_{EC3}	A 692	A 700	A 698	A 699
W_{EC4}	A 743	A 736	A 743	A 740
W_{EC5}	A 459	A 434	A 442	A 440

Table S5 Distance geometry (Å) of CO_2, HCO_3^- and key bound water molecules in the native and V143I CA II.

	native 0atm 6KM3	native 7atm 6KM4	native 13atm 6KM5	native 15atm 6KM6	V143I 0atm 6KLZ	V143I 7atm 6KM0	V143I 13atm 6KM1	V143I 15atm 6KM2
Zn – W_{Zn}	1.90	1.93	1.93	1.93	1.89	1.89	1.93	1.92
Zn – W_{DW}	4.14	–	–	–	4.09	4.12	3.07	3.07
Zn – CO_2(O1)	–	3.23	3.27	3.28	–	–	–	–
Zn – HCO_3^-(O1)	–	–	–	–	3.17	2.92	3.00	3.01
Zn – HCO_3^-(O3)	–	–	–	–	1.99	1.98	1.99	1.99
W_{Zn} – W_{DW}	2.51	–	–	–	2.61	2.75	–	–
W_{Zn} – CO_2(O1)	–	2.89	2.92	2.94	–	–	2.92	2.90
W_{Zn} – CO_2(O2)	–	3.07	3.06	3.07	–	–	2.90	2.88
W_{Zn} – CO_2(C)	–	2.79	2.78	2.79	–	–	2.68	2.67
W_{Zn} – HCO_3^-(O1)	–	–	–	–	2.32	2.27	2.32	2.29
W_{Zn} – HCO_3^-(O2)	–	–	–	–	2.63	2.51	2.43	2.44
W_{Zn} – HCO_3^-(O3)	–	–	–	–	0.31	0.60	0.69	0.67
W_{Zn} - HCO_3^-(C)	–	–	–	–	1.56	1.48	1.49	1.50
Val143(Cγ2) – W_{DW}	5.28	–	–	–	–	–	–	–
Val143(Cγ1) – W_{DW}	5.80	–	–	–	–	–	–	–
Val143(Cγ2) – CO_2(O1)	–	4.01	3.93	3.94	–	–	–	–
Val143(Cγ2) – CO_2(O2)	–	4.19	4.16	4.15	–	–	–	–
Val143(Cγ2) – CO_2(C)	–	3.93	3.87	3.87	–	–	–	–
Val143(Cγ1) – CO_2(O1)	–	3.73	3.68	3.70	–	–	–	–
Val143(Cγ1) – CO_2(O2)	–	4.75	4.77	4.79	–	–	–	–
Val143(Cγ1) – CO_2(C)	–	4.13	4.11	4.12	–	–	–	–
Ile143(Cδ1) – W_{DW}	–	–	–	–	3.53	3.41	–	–
Ile143(Cγ2) – W_{DW}	–	–	–	–	5.41	5.23	–	–
Ile143(Cγ1) – W_{DW}	–	–	–	–	5.04	4.89	–	–
Ile143(Cδ1) – CO_2(O1)	–	–	–	–	–	–	3.17	3.17
Ile143(Cδ1) – CO_2(O2)	–	–	–	–	–	–	3.23	3.20
Ile143(Cδ1) – CO_2(C)	–	–	–	–	–	–	2.97	2.95
Ile143(Cγ2) – CO_2(O1)	–	–	–	–	–	–	3.77	3.78
Ile143(Cγ2) – CO_2(O2)	–	–	–	–	–	–	5.04	5.01
Ile143(Cγ2) – CO_2(C)	–	–	–	–	–	–	4.29	4.28
Ile143(Cγ1) – CO_2(O1)	–	–	–	–	–	–	4.25	4.23
Ile143(Cγ1) – CO_2(O2)	–	–	–	–	–	–	4.73	4.68
Ile143(Cγ1) – CO_2(C)	–	–	–	–	–	–	4.34	4.29
Ile143(Cδ1) – HCO_3^-(O1)	–	–	–	–	3.78	3.73	3.86	3.86
Ile143(Cδ1) – HCO_3^-(O2)	–	–	–	–	4.08	4.08	4.30	4.25
Ile143(Cδ1) – HCO_3^-(O3)	–	–	–	–	5.19	5.08	5.14	5.11
Ile143(Cδ1) – HCO_3^-(C)	–	–	–	–	4.20	4.17	4.28	4.23
Ile143(Cγ2) – HCO_3^-(O1)	–	–	–	–	5.05	4.81	4.97	5.00
Ile143(Cγ2) – HCO_3^-(O2)	–	–	–	–	6.16	6.03	6.17	6.14

Ile143(Cγ2) – HCO_3^-(O3)	–	–	–	–	6.05	5.84	5.84	5.83
Ile143(Cγ2) – HCO_3^-(C)	–	–	–	–	5.67	5.43	5.53	5.50
Ile143(Cγ1) – HCO_3^-(O1)	–	–	–	–	5.01	4.94	5.12	5.10
Ile143(Cγ1) – HCO_3^-(O2)	–	–	–	–	5.59	5.58	5.83	5.76
Ile143(Cγ1) – HCO_3^-(O3)	–	–	–	–	6.50	6.37	6.44	6.39
Ile143(Cγ1) – HCO_3^-(C)	–	–	–	–	5.61	5.54	5.68	5.61
W_{EC2}'''' – HCO_3^-(O1)	–	–	–	–	1.38	–	–	–
W_{EC2}'''' – HCO_3^-(O2)	–	–	–	–	2.51	–	–	–
W_{EC2}'''' – HCO_3^-(O3)	–	–	–	–	3.46	–	–	–
W_{EC2}'''' – HCO_3^-(C)	–	–	–	–	2.30	–	–	–
W_{EC2}' – HCO_3^-(O1)	–	–	–	–	–	2.73	2.66	2.62
W_{EC2}' – HCO_3^-(O2)	–	–	–	–	–	3.56	3.67	3.66
W_{EC2}' – HCO_3^-(O3)	–	–	–	–	–	4.31	4.42	4.40
W_{EC2}' – HCO_3^-(C)	–	–	–	–	–	3.46	3.52	3.50
W_I – CO_2(O1)	–	3.89	3.97	4.02	–	–	3.85	3.83
W_I – CO_2(O2)	–	3.29	3.31	3.34	–	–	3.01	3.03
W_I – CO_2(C)	–	3.42	3.47	3.51	–	–	3.26	3.26
W_I – HCO_3^-(O1)	–	–	–	–	2.29	2.72	2.70	2.66
W_I – HCO_3^-(O2)	–	–	–	–	1.44	1.68	1.79	1.82
W_I – HCO_3^-(O3)	–	–	–	–	2.47	2.73	2.87	2.86
W_I – HCO_3^-(C)	–	–	–	–	1.61	2.12	2.19	2.20
W_{EC2}' – CO_2(O1)	–	3.73	3.78	3.79	–	–	3.66	3.60
W_{EC2}' – CO_2(O2)	–	4.41	4.42	4.42	–	–	4.20	4.19
W_{EC2}' – CO_2(C)	–	3.94	3.95	3.94	–	–	3.76	3.72
W_{EC2} – W_{DW}	4.39	–	–	–	–	–	–	–
W1 – W_{Zn}	2.64	–	–	–	2.60	2.59	2.56	2.57
W_I – W_I'	–	2.09	2.12	2.11	–	2.08	2.06	2.14
W2 – W_{Zn}	–	4.67	4.68	4.68	–	–	–	–
W_{EC2}'''' – W_I	–	–	–	–	2.43	–	–	–
W_{EC2}'''' – W1	–	–	–	–	3.28	–	–	–
W_{EC2}'''' – W_{EC2}	–	–	–	–	2.07	–	–	–
W_{EC2}'''' – W_{EC2}''	–	–	–	–	3.24	–	–	–
W_{EC2}'''' – W_{EC2}'''	–	–	–	–	1.71	–	–	–
W_{EC2} – W_I	–	–	–	–	3.13	3.28	3.20	3.16
W_{EC2} – W1	2.79	–	–	–	2.78	2.62	2.64	2.65
W_{EC2}' – W_I	–	3.15	3.16	3.15	–	2.94	2.98	2.96
W_{EC2}' – W1	–	–	–	–	–	3.04	3.14	3.12
W2 – W_I	–	4.71	4.71	4.73	4.78	4.78	4.62	4.62
$His64_{in}$(Nδ1) – W2	3.24	3.30	3.25	3.23	3.29	3.40	3.32	3.35
$His64_{in}$(Nδ1) – W2′	–	1.82	1.75	1.76	–	1.76	1.93	1.92

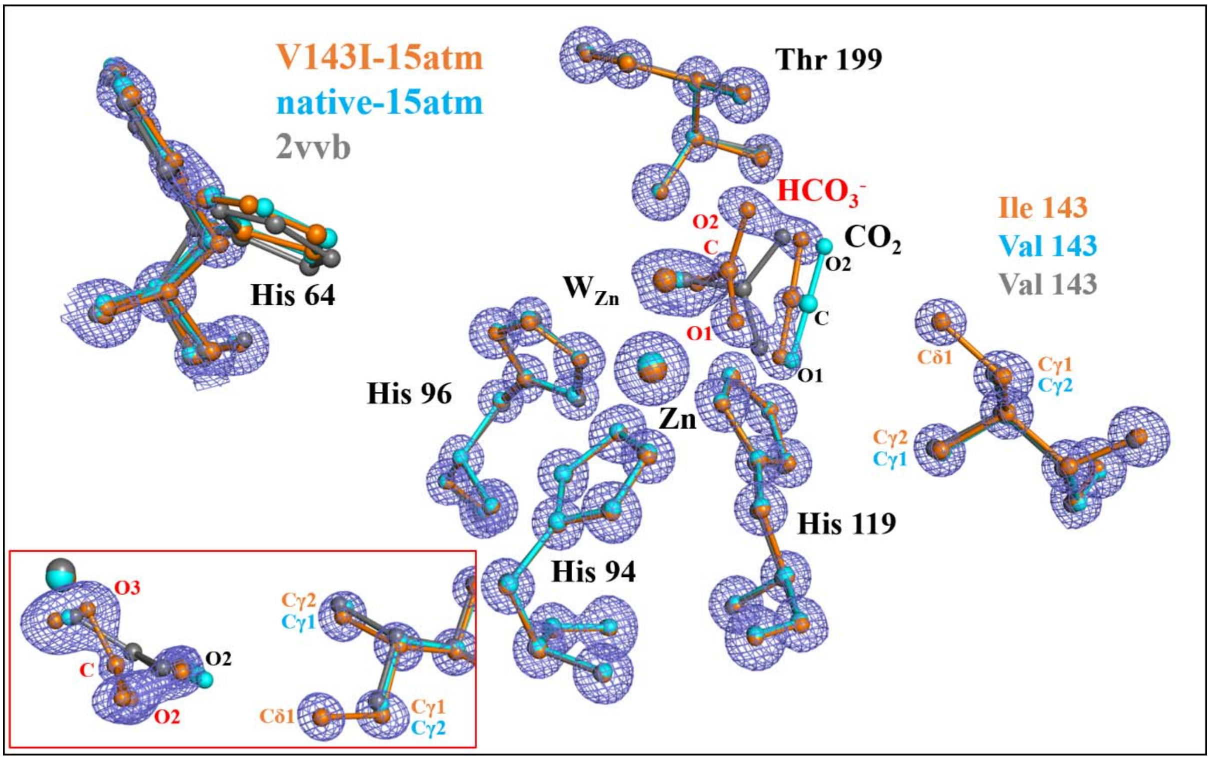

Figure S1 Close view of the active site of V143I-15atm (orange, 0.9 Å resolution). The structure of native-15atm (cyan, 1.15 Å resolution) and the structure of the native CA II with HCO_3^- (grey, 1.66 Å, PDB code: 2VVB) are superimposed for comparison. (Sjoeblom *et al.*, 2009) The active site water network (W_I/W2/W3a/W3b) is not shown for clarity. The electron density map is from V143I-15 atm. The 2Fo-Fc map is contoured at 4.0 σ, except His64 which is at 2.5 σ, and W_{Zn}, CO_2, and HCO_3^- at 2.0 σ. The atom names are labelled for CO_2 (black), HCO_3^- (red), Isoleucine 143 (orange) and Valine 143 (cyan). The inset (red box) shows the CO_2 and HCO_3^- at different angle. Note that the W_{Zn}, CO_2 and HCO_3^- in native CA II are roughly within a plane. On the other hand, V143I-15atm shows that both CO_2 and O1 and O2 atoms of HCO_3^- are pushed and twisted toward W_{Zn} and O3 atom of HCO_3^- is pushed away from W_{Zn}. This movement results in HCO_3^- being displaced from the plane defined by W_{Zn}, CO_2 and HCO_3^- in the native CA II form.

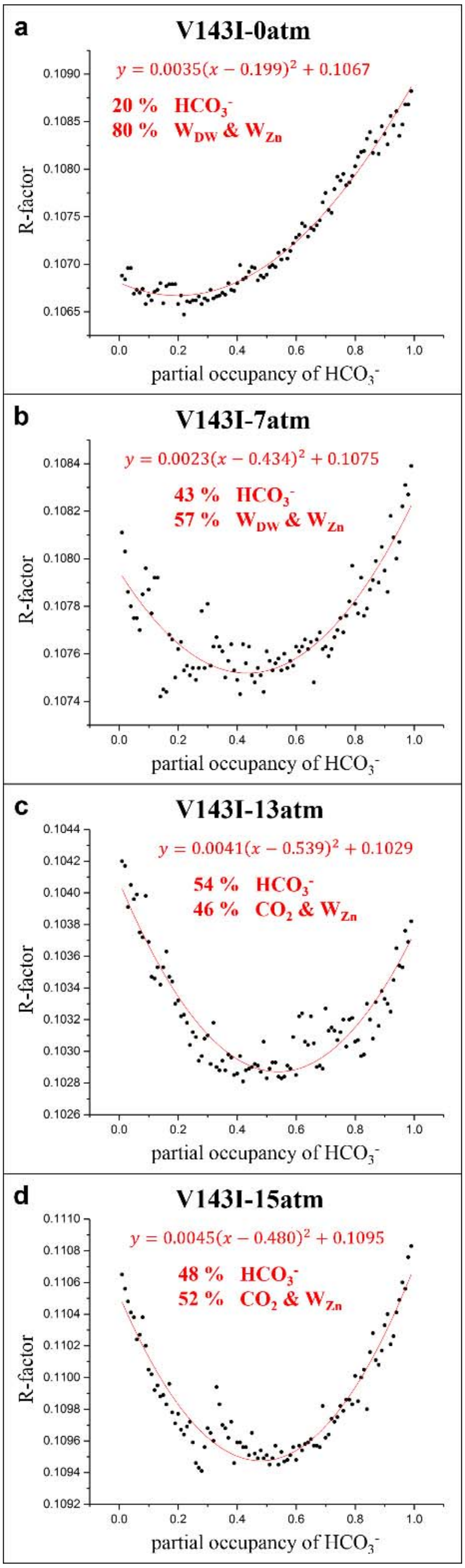

Figure S2 Partial occupancy determination of HCO_3^- in V143I CA II **(a-d)**. For each data set, systematic refinements were carried out on 99 structures with manually adjusted HCO_3^-/CO_2/W_{Zn}/W_{DW} occupancies. The obtained data points were then fitted to quadratic functions, showing the minimum points in the overall R factors.

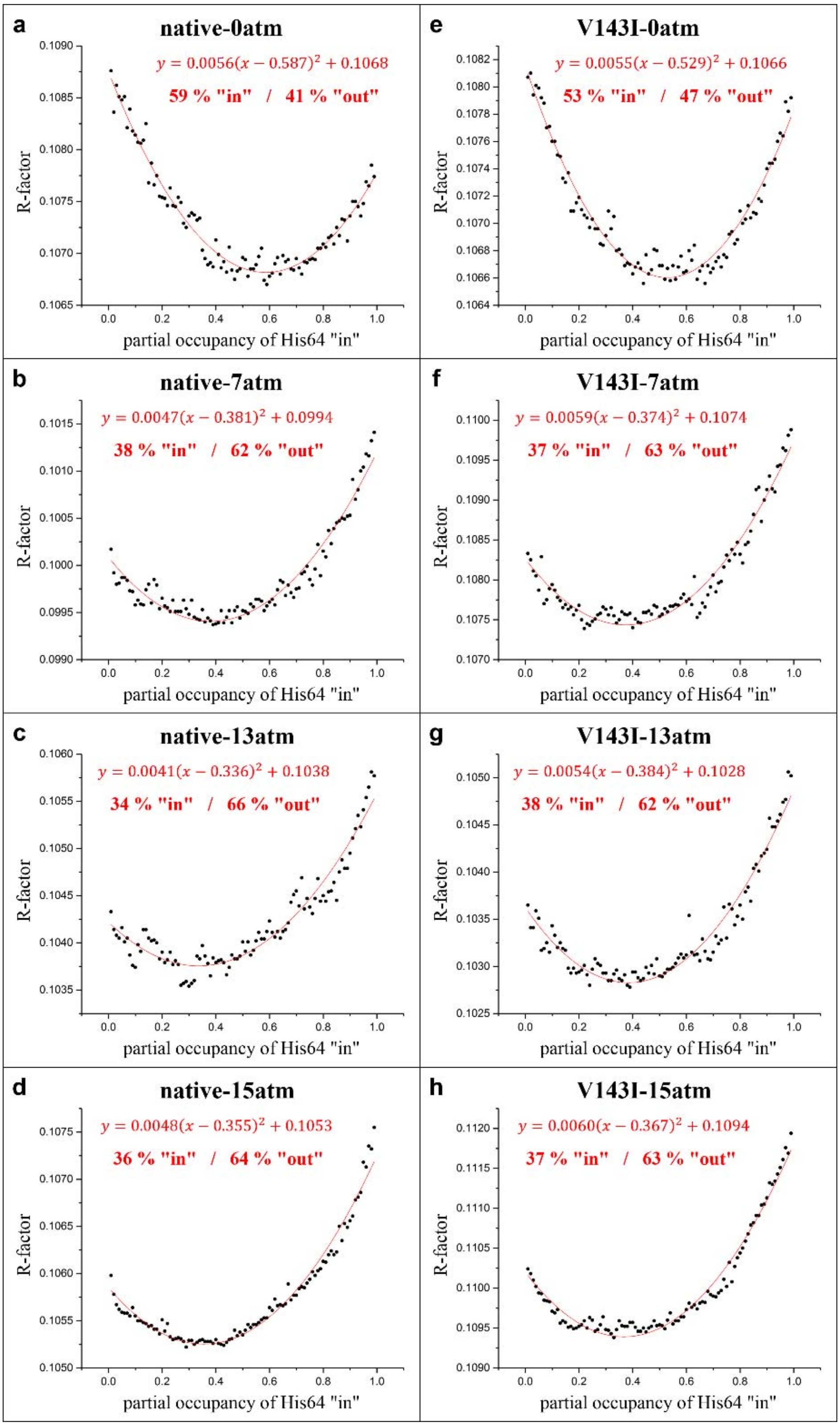

Figure S3 Partial occupancy determination of His64 in native CA II **(a-d)** and V143I CA II **(e-h)**. For each data set, systematic refinements were carried out on 99 structures with manually adjusted His64 in/out occupancies. The obtained data points were then fitted to quadratic functions, showing the minimum points in the overall R factors.
